# Comparative study of lung cancer care and survival outcomes across the Nordic countries

**DOI:** 10.2340/1651-226X.2025.42778

**Published:** 2025-06-04

**Authors:** Ghida Khalife, Juho Warisa, Uffe Bødtger, Johan Isaksson, Kirill Neumann, Hrönn Harðardóttir, Heidi Andersén, Antti Jekunen, Maria Lovén, Tuula Vasankari, Susanna Nurmi-Rantala, Paulus Torkki

**Affiliations:** a Department of Public Health, University of Helsinki, Helsinki, Finland; b Respiratory Research Unit PLUZ, Department of Internal and Respiratory Medicine, Zealand University, Hospital Roskilde & Nastved, Næstved, Denmark; c Institute of Regional Health Research, University of Southern Denmark, Odense, Denmark; d Centre for Research and Development, Gävle County/Uppsala University, Uppsala, Sweden; e Pulmonary Department, Akershus University Hospital, Oslo, Norway; f Department of Respiratory Medicine, Landspitali University Hospital, Reykjavik, Iceland; g Cancer Clinic, Vaasa Central Hospital, Vaasa, Finland; h Finnish Lung Health Association (FILHA), Helsinki, Finland; i Faculty of Medicine, Oncology Department, University of Turku, Turku, Finland; j Faculty of Medicine and Health Technology, Tampere University, Tampere, Finland; k Department of Pulmonary Diseases and Clinical Allergology, University of Turku, Turku, Finland; l MSD Finland, Espoo, Finland

**Keywords:** Lung cancer, care practices, treatment outcomes, survival rate, Nordic countries

## Abstract

**Background:**

Lung cancer (LC) is the leading cause of cancer-related deaths worldwide. Despite societal, economic and genetic similarities, 5-year age-standardized relative survival rate is lower in Finland compared to the other Nordic countries. Previous studies have identified discrepancies in LC guidelines, but research on actual care practices remains limited. We aim to address this knowledge gap by conducting a comprehensive examination of the current care practices for LC patients in the Nordic countries.

**Methods:**

We employed a non-interventional, prospective study design. We conducted an expert workshop involving LC specialists from Finland to formulate relevant questions for a structured survey. This survey was distributed to healthcare professionals (HCPs) across Nordic hospitals and primary care units. The survey results were then analyzed, and a follow-up Nordic LC expert workshop was held to identify the most relevant factors potentially influencing LC survival outcomes.

**Results:**

Four key differences in care practices between Finland and other Nordic countries were identified: (1) resources available in primary care units, (2) waiting times in primary care, (3) availability of novel treatments and (4) tracking of LC survival and mortality outcomes by the hospital. Finland has the lowest access to computed tomography (CT) from primary care, longest waiting times in primary care, and lacks a national outcome tracking system. Some medical doctors in Finland and Iceland highlighted observed limitations in specific cases involving access to neoadjuvant immunotherapy and chemotherapy.

**Interpretation:**

Several factors unrelated to specialized LC care are likely contributing to poorer 5-year survival rates for LC in Finland. These findings may be applicable to other healthcare systems as well.

## Introduction

In 2020, an estimated 2.2 million new lung cancer (LC) cases were reported globally, making it the second most diagnosed cancer [1]. The high incidence combined with a high mortality rate, approximately 1.8 million LC-related deaths globally in that year, makes it the leading cause of cancer-related mortality worldwide [1]. Despite similar LC incidence rates to other Western countries, the Nordic countries (Denmark, Finland, Iceland, Norway, Sweden), have relatively high survival rates [2, 3]. These countries are among the world’s richest countries, with profound societal and political homogeneity, free access to healthcare and unique opportunities for joint health registry-based research [4]. Their similarly organized healthcare systems contribute to minimal differences in survival rates for most cancers [5].

LC patients’ 1- and 5-year age-standardized relative survival rates depend on many factors [6–8]. The most important factor influencing the survival rate is the stage at diagnosis. He et al. reported the relative 5-year LC specific survival rate for stage I patients at 82.3% and 31% for stage IV [8]. The stage at diagnosis also determines the possibility to receive surgical resection, which has been shown to influence the survival outcomes [9, 10]. The histological type and the molecular subtype of cancer also influence survival rates. Squamous cell carcinoma and small cell carcinoma are associated with lower survival rates compared to adenocarcinoma [8]. Factors such as smoking significantly increase the proportion of squamous cell carcinoma and small cell carcinoma among LC cases [11]. Sex, age, and performance status are also important determinants of survival, with men demonstrating lower age-standardized 1- and 5-year survival compared to women across the Nordic countries. Increasing age and poorer performance status are further associated with reduced survival [12, 13]. Lastly, timely access to appropriate treatment and high-quality healthcare services is a critical determining factor of survival outcome [14].

Since the early 2000s, Finland’s 1- and 5-year age-standardized survival rates for LC in both men and women have improved more slowly compared to other Nordic countries. For instance, in 1998–2002, Finland’s 5-year survival rate for men was 9.7%, close to Denmark’s lowest rate (8.9%) and Iceland’s highest (11.9%). By 2018–2022, Finland reported the lowest survival rate (16.7%), significantly trailing Denmark (26.2%) and Iceland (28.4%) [12].

Patients’ characteristics are broadly similar across the Nordics. According to recent studies, LC patients’ performance status scores are comparable between the countries. In Sweden, Denmark, and Finland, 65–70% of patients were classified as ECOG performance status 0–1, while in Norway the corresponding figure was 50.5% [15–17]. However, these figures should be interpreted with caution, as performance status data were missing for 70% of patients in Finland and 23.4% in Norway [15, 17]. Regarding age distribution, 2022 data show that LC incidence in both males and females in the Nordic countries increases sharply after the age of 50, with broadly similar patterns across countries. In most cases, incidence peaks between ages 75 and 85, although among males in Iceland, the peak occurs at a slightly older age [12]. Recent data on the gender distribution of LC patients show that the proportion of male patients in Finland (60%) is higher than in other Nordic countries, including Norway (51%), Sweden (45%), Denmark (49%), and Iceland (46%) [12]. NORDCAN provides limited data on the distribution of cancer subtypes by country. However, the trends in cancer subtypes appear to be similar across countries, with an increasing proportion of adenocarcinomas over time [15, 18–20]. The reporting of cancer stages in NORDCAN has been inconsistent and methods for coding disease extent have varied over time and between countries [21]. However, recent studies indicate that the proportion of early-stage diagnoses in Finland is lower than that observed in Denmark and Norway [15, 22].

Although country-specific differences in the distribution of cancer stages and histological types may partially explain differences in LC survival, Tichanek et al. propose that disparities in healthcare resources are likely to contribute to the observed differences in LC survival in the Nordic region. However, research comparing healthcare systems through integrated analyses of practices and survival outcomes remains limited. Most comparative studies have focused on individual aspects, such as health policies [23], practices or guidelines [22, 24], or outcome measures [14, 25, 26] separately.

Our study aims are (1) determining the current care practices for LC patients in the Nordic countries and (2) identifying the factors that are most likely to cause differences in survival outcomes of LC patients.

## Research design and methodology

This study aims to determine the current LC care practices in Nordic countries through a non-interventional survey study. We conducted the study in three phases: (1) a national expert workshop defining the survey questions, (2) a structured survey for the healthcare professionals (HCPs) and (3) a Nordic expert’s workshop to assess the results.

### Phase 1: Finnish expert workshop

The main objective of this workshop was to gather expert opinions on the most relevant factors affecting LC care to formulate the survey questions for Nordic HCPs. In the online workshop, HCPs (*n* = 6) from primary and secondary care in Finland (the Finnish experts) discussed the clinical practices in all phases of care: primary care, diagnostic, treatment, and follow-up phase. During the workshop, the facilitator (P.T.) steered the discussion and recorded the main points, with the contribution of the research group (G.K. & J.W.).

The participants were representatives of the National Lung Cancer Program in Finland, initiated by Finnish Lung Health Association. They were selected to ensure both geographic and institutional diversity, representing five major healthcare districts across the country. The group included professionals from various points along the LC care pathway, such as an oncologist, thoracic surgeon, pulmonologist, radiologist, and a primary care physician.

### Phase 2: Structured survey

#### Survey design and distribution

The structured survey was finalized in collaboration with the Finnish experts and had a total of 34 questions (Supplementary Materials). The final survey was constructed and distributed through the REDCap electronic data tool [27].

A participant list was created (*n* = 127) including HCPs from hospitals and primary care units from each of the Nordic countries. The list was compiled using information from hospital websites, professional medical associations, and national healthcare directories. Participants included oncologists, pulmonologists, thoracic surgeons, and general practitioners (GPs), with the main inclusion criteria being (1) HCPs responsible for treating LC patients in the Nordic countries and (2) agreement to participate in the study. The selection process targeted various regions within each country.

While the survey was anonymous, regional and institutional coverage was achieved by utilizing national networks and targeting key centers. From the 58 healthcare regions in the Nordic countries, responses were received from 30 regions. Hospital representation included 21 from university hospitals, 5 from regional hospitals, and 6 from central hospitals.

### Ethical considerations

All survey participants consented to answering the survey. They were informed about the study’s purpose, data usage, and privacy measures. No personal or patient-specific data were collected, so IRB/EC review and informed consent were not required.

### Data analysis

Numerical values were analyzed by considering the average values, deviations and distributions. Due to the nature of the questions and low number of responses, statistical significances were not analyzed.

### Phase 3: Nordic Expert Workshop

After analyzing survey results, the final phase of this study involved a Nordic expert workshop online. The workshop’s main objective was to assess whether the survey results reflect the actual practices in each country.

Ten participants were invited to attend the workshop from all Nordic countries (the Nordic experts). They represented diverse expertise and included thoracic surgeons, a primary care physician, pulmonologists, and oncologists. The workshop slides, containing all results, were distributed in advance to allow participants time to review the data and prepare their insights.

Experts were selected based on the following inclusion criteria: (1) responsibility for treating LC patients (ICD-10 C34), (2) agreement to participate, and (3) a minimum of 5 years of experience. Most were affiliated with university hospitals or national research centers, bringing substantial clinical and policy-level experience.

The workshop focused separately on each phase of the LC care pathway (primary care, diagnostic, treatment, and follow-up phase) and the insights of Nordic experts were analyzed as representing their countries. Participants shared their final thoughts, focusing on which differences they believed most likely explained the variations in LC survival between the Nordic countries. The facilitator (P.T.) presented the survey findings and steered the conversation. A summary report including all key discussion points was sent to the participants.

All data collected during the survey and workshop were handled in accordance with the General Data Protection Regulation and relevant EU regulations to ensure the privacy and confidentiality of participants.

## Results

### Demographics and respondents

Of the 52 individuals who participated, 42 responses were included in the analysis. Ten responses were excluded since they were incomplete. Among the 42 respondents, 21 mentioned working in a hospital, 10 stated working in a primary care unit, and 11 reported working in both a hospital and a primary care unit (Table 1). The respondents included a mix of relevant specialities.

**Table 1:** Number of survey respondents in each country and number of healthcare regions covered in each country.

Country	Respondents from hospitals	Respondents from primary care units	Respondents representing both a hospital and a primary care unit	Healthcare regions covered in the responses out of total number of healthcare regions in each country
Finland	4	4	4	8/21
Sweden	7	2	2	8/21
Denmark	6	2	0	4/5
Norway	4	1	3	4/4
Iceland	0	1	2	1/7
Total	21 (22)	10 (16)	11	30/58

### Waiting time

Throughout the LC care pathway, waiting time was longest in Finland (Table 2). Other countries had only minor differences in waiting times. The Finnish experts observed that the average 14-day waiting time reported in the survey for scheduling an appointment at a primary care unit for patients exhibiting LC symptoms might be optimistic. They elaborated that it often takes up to a month to secure such appointments in numerous Finnish regions. The Nordic experts concurred that a few weeks difference in waiting times does not explain the difference in LC survival.

**Table 2:** Waiting times in different stages of LC treatment pathway and the share of patients meeting a doctor in the contact in primary care per country.

Mode of reported waiting time	Finland	Sweden	Denmark	Norway	Iceland
To secure an appointment at the health center for patients with LC symptoms (days)	14	2–7	2–7	2–7	2–7
For specialist consultation after initial visit to a GP (weeks)	2–4	1–2	1–2	1–2	2–3
For a LC diagnosis after specialist consultation (weeks)	4	4	2–4	2–4	1–2
Mode of reported share of patients meeting a doctor in the first healthcare contact	40–60%	80–100%	100%	100%	100%

LC: lung cancer; GP: general practitioner.

### Meeting a GP at first primary care visit

The ratio of patients meeting a GP on their first primary care visit was lowest in Finland (Table 2). In Sweden, one respondent reported that 80% of the patients meet a doctor, while others stated that all patients do. In both Finland and Sweden, patients who do not meet a GP on first contact are seen by a nurse. In contrast, in Norway, Denmark, and Iceland, all patients reportedly see a doctor during their first contact.

### Access to imaging in primary care

**Figure 1:**
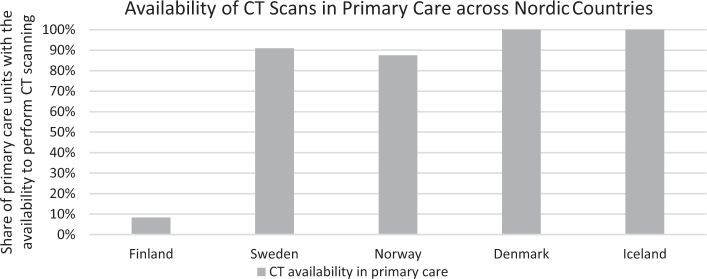
The share of HCPs from each Nordic country who reported the presence of CT scan facilities in primary care settings.

The availability of diagnostic imaging modalities in primary care was uniform across the Nordic countries except for CT scanning. Only one respondent from Finland reported that they have CT scan as an imaging option in primary care in the region. In other Nordic countries, CT scanning was more readily available (Figure 1). The Nordic experts agreed that the availability of CT scanning in primary care is likely to affect LC survival.

### LC treatment

During the treatment phase, the availability of treatment options for LC was largely similar across the Nordic countries. However, small country-specific differences were reported in the availability of novel treatments. Among respondents from specialized healthcare, three out of eight in Finland, one out of two in Iceland, and two out of seven in Norway expressed the opinion that decisions made within the public healthcare system limit some patients’ access to optimal LC medications. In Iceland, this was described more as an observed limitation in specific cases involving neoadjuvant immunotherapy and chemotherapy. In contrast, all Swedish and Danish respondents indicated that such decisions do not restrict access to medications. Finnish and Icelandic respondents further specified that these restrictions primarily target novel treatments.

### Survival outcome tracking by the hospital

All Finnish and Icelandic respondents reported that their hospitals do not track LC treatment outcomes (survival and mortality) (Table 3). Three out of seven respondents in Norway reported that their hospitals do not track treatment outcomes, two reported that outcomes are discussed systematically and regularly and two were uncertain. In Sweden and Denmark, all respondents except one reported that outcomes are either only measured or measured and discussed systematically and regularly.

**Table 3:** The mode of outcome tracking practices in Nordic countries.

	Finland	Sweden	Denmark	Norway	Iceland
National quality register to track outcomes	No	Yes	Yes	Yes	Yes
Tracking outcomes by hospital	Not systematically tracked	Discussed regularly and systematically by the hospital	Discussed regularly and systematically by the hospital	Not systematically tracked	Not systematically tracked

Survey questions:

1. Is there a national quality register specifically for tracking LC treatment outcomes?

2. Does your hospital track the outcomes of LC patients (e.g., mortality, survival rate)?

## Discussion

This study identified key differences in LC care practices among the Nordic countries that may collectively contribute to Finland’s lower 5-year age-standardized survival rate for LC patients. These differences primarily concern the availability and use of CT scanning in primary care, longer waiting times throughout the LC care pathway, and the difference in initial appointments, where patients in Finland are more likely to meet nurses rather than doctors compared to other Nordic countries. Collectively, these factors increase the likelihood of diagnostic delays, resulting in later-stage diagnoses and, consequently, poorer survival outcomes.

### CT scanning and early diagnosis

The most notable difference was Finland’s lack of CT scanning usage in primary care for patients with LC related symptoms. In Finland, CT scans are typically only accessible after referral to secondary care, often following a suspicious chest X-ray finding. However, chest X-rays are less sensitive in detecting small tumors and prone to false-negative results [28]. This can potentially lead to significant delays in diagnosis, especially in early-stage LC, which have significantly better survival rates compared to more progressed disease [7]. This reliance on chest X-rays may partly stem from Finland’s healthcare resource constraints. Among the Nordic countries, Finland has relatively lower healthcare funding and the fewest CT scanners per 100,000 inhabitants, which could limit the accessibility of advanced imaging technologies in primary care settings [29]. These resource limitations likely contribute to delays in early-stage LC detection [3]. This is reflected in the comparatively low proportion of early-stage diagnoses in Finland, where only 17% of patients are diagnosed at stage I, compared to over 25% in Denmark and Norway [15, 22]. Nonetheless, stage at diagnosis is inconsistently recorded in Finnish cancer registries [22], which may indicate even greater underdetection of early-stage disease.

The Nordic experts acknowledged that the differences in access to CT scanning in primary care may influence differences in LC prognosis. Finland’s comparatively limited availability of CT scans presents challenges for early detection, which is critical for improving outcomes. This is in accordance with results from The National Lung Screening Trial (NLST), where the use of CT screening was related to better survival compared to chest x-ray screening [30].

### Delayed access to care

Another key factor is the extended waiting time in Finland across the LC care pathway. National waiting time data supports Finnish experts’ notion that our results regarding waiting times in Finland might be optimistic. In 2023, 35% of patients waited longer than 14 days to see a doctor in primary care, and 14% waited for 1–3 months [31]. Although these figures are not specific to LC patients, LC symptoms are often subtle and non-specific (e.g., increased cough, breathlessness) [32], making expedited appointments less likely. Prolonged waiting times may increase patients’ thresholds for seeking care, further compounding delays in diagnosis and treatment [33]. Besides, it was found in the survey that patients in Finland are more likely to initially encounter a nurse rather than a physician during their first primary care contact. While this alone may not directly cause delays, it may reflect broader systemic characteristics of Finland’s strong gatekeeping model. Previous studies have shown that healthcare systems with strong primary care gatekeeping, such as Finland’s, are often associated with longer diagnostic intervals for cancer [34]. This suggests that delays may be more attributable to the structure and functioning of the healthcare system than to the specific professional seen at first contact.

The significance of these delays remains debated. While the Nordic experts believed short delays might have minimal impact on survival, the literature presents mixed evidence. Some studies suggest that longer waiting times are associated with better outcomes, possibly because patients with more severe symptoms receive prioritized care [35–37]. Conversely, other studies emphasize the adverse effects of delays, especially for early-stage LC. Khorana et al. [38] found that each week of treatment delay increased the risk of death by 3.2% for stage I and 1.6% for stage II LC. Similarly, O’Rourke et al. [39] demonstrated that even short treatment delays could lead to substantial tumor growth, potentially rendering curable cancers incurable. These findings suggest that the cumulative 1–2 month delay in Finland, as reported in our survey and by the Finnish Institute for Health and Welfare [31], may have a notable impact on survival outcomes.

### Treatment access barriers

Beyond individual patient delays, systemic factors may also hinder access to novel treatments in Finland. While approval timelines are comparable to other Nordic countries, the decentralized financial structure in Finland means that hospital-administered drugs often require case-by-case funding decisions at the hospital level. This contrasts with national procurement and reimbursement systems in countries such as Sweden and Norway, which may allow for faster and more consistent uptake. Such structural barriers may contribute to slower adoption and regional variation in access to new cancer therapies in Finland [40].

### Lack of outcome tracking

In addition, the systematic use of survival and mortality outcome information may be linked to the development of these outcomes. In Sweden and Norway, hospitals systematically utilize outcome data to refine care processes, professional practices, and education. This approach enables the identification of gaps and the implementation of incremental changes that collectively enhance patient care. A review by Bhati et al. highlights the important role of hospital administration in influencing patient outcomes through data-driven performance measurement [41]. Thus, while many Nordic countries rely on Cancer Registry data for long-term survival tracking, integrating hospital-based outcome tracking alongside registry data could complement this by providing a more immediate feedback mechanism to support continuous improvements in treatment quality and processes.

Therefore, Finland’s lack of CT scanning in primary care, longer waiting times, and differences in initial appointments likely contribute to delayed LC diagnosis in Finland, resulting in more late-stage diagnoses and survival disparities. While definitive proof of this mechanism is lacking, these findings highlight the need for developing the quality registries to enable the detailed comparison between countries.

### Global relevance

Significant differences in LC survival outcomes exist across healthcare systems worldwide [1]. By applying the methodologies and approaches used in our study, other regions can identify factors associated with these differences. This can potentially inform and enhance LC care practices globally, showing that our findings are not only applicable in Finland but also in diverse international contexts.

### Strengths and limitations

This study’s strength lies in the strong participation of 42 HCPs from both primary and secondary care, covering over half of the Nordic healthcare regions and various specialties in LC care. In addition, workshops included specialists from different phases of the LC care pathway and representatives from all Nordic countries who evaluated the reliability of the survey results.

A limitation of this study is the variability in cancer registration practices across the Nordic countries, which affects the comparability of survival statistics [22]. For instance, 1-year age-standardized relative survival estimates in Sweden are likely overestimated by 2–3 percentage points because the Swedish Cancer Registry excludes death-certificate-initiated cancers, which typically involve short survival times [21]. However, since we analyzed 5-year age-standardized survival rates using longitudinal data for all countries and validated the results with the Nordic experts, we expect this issue to have minimal impact on our findings.

We opted to create our own survey, with the input from the Finnish experts, to tailor the questions specifically to our research objectives and the unique context of the Nordic countries. This approach allowed us to gather more relevant and targeted data, despite the potential limitations associated with using a non-validated survey method.

This study may overlook regional variation, as the findings might not fully represent practices across the entire country. The study’s reliance on a small number of respondents per country (fewer than 11) is mitigated by the validation from the Nordic expert workshop. Nonetheless, the findings may reflect the influence of confounders such as professional background, institutional practices, geographic differences, and personal biases.

Lastly, establishing accurate relationships between the identified practice differences and outcomes is limited, which restricts the study’s ability to draw definitive conclusions. Therefore, the differences identified as potentially impacting survival rates remain hypothetical and require further examination.

## Conclusions

Differences in timely access to healthcare and early detection may help explain the survival rate disparities in LC between Finland and the other Nordic countries. While the development of healthcare systems often prioritizes new technologies, a more effective approach may be to adapt existing best practices and encourage knowledge-sharing among countries with similar systems. Although the differences in care practices discussed in this study may contribute to variations in survival rates, their causal relationships and precise impact require further investigation.

## Supplementary Material

Comparative study of lung cancer care and survival outcomes across the Nordic countries

## Data Availability

The data supporting this study were collected through the REDCap platform. Due to ethical and privacy considerations, the full dataset is not publicly available. However, aggregated data or limited access may be provided upon reasonable request and pending approval from the relevant ethical review board. For inquiries, please contact ghida.khalife@helsinki.fi.
