# Supplementary material for: Comparative study of lung cancer care and survival outcomes across the Nordic countries
Source: Acta Oncol. 2025 Jun 4;64:42778. doi: 10.2340/1651-226X.2025.42778 (PMC12160590; doi:10.2340/1651-226X.2025.42778)
Supplement: Comparative study of lung cancer care and survival outcomes across the Nordic countries [file AO-64-42778-s1.pdf]

Supplementary material has been published as submitted. It has not been copyedited, or typeset by Acta Oncologica

Supplementary Material 1. Structured Survey

Hospital Option:

Hospital type:
a) University hospital
b) Central hospital
c) Regional hospital

Catchment area of the hospital (Number of residents):

d) More than 500 000 people
e) 200 000 – 499 999 people
f) 100 000 – 199 999 people
g) <100 000 people

Health Center Option:

Catchment area of the health center (Number of residents):
a) Less than 10,000
b) 10,000-50,000
c) 50,000-100,000
d) 100,000-500,000
e) More than 500,000

What is the typical waiting time for patients experiencing lung cancer-like symptoms to secure an appointment at the health center?
a) More than 4 weeks
b) 2-4 weeks
c) 1-2 weeks
d) 2-7 days
e) Less than 2 days

Which option describes best the share of doctor / nurse visits in first contacts:
a) All patients meet doctor
b) 80 % of the patients meet doctor, and the others meet nurse
c) 60 % of the patients meet doctor, and the others meet nurse
d) 40 % of the patients meet doctor, and the others meet nurse
e) less than 40 % of the patients meet doctor
f) patients meet typically both doctor and nurse during the visit
g) I'm not sure

A. Primary Care Phase

1) What are the symptoms that prompt suspicion of lung cancer?
   - Persistent cough
   - Shortness of breath
   - Chest pain
   - Fatigue
   - Unintentional weight loss
   - Hemoptysis
   - Reduced lung sounds
   - Other (please specify)
   - I'm not sure

2) What types of patients are typically referred for further lung cancer evaluation and treatment? (Select one or multiple)
- Patients with a history or family history of smoking
- Patients with a history or family history of cancer
- Patients with symptoms suggestive of lung cancer
- Patients with heavy smoking history and lung cancer symptoms
- Patients with abnormal chest imaging results
- Older age
- Others (Please specify)
- I'm not sure

3) Is lung cancer screening being utilized, and if so, to what extent?
- Yes, and it is offered to high-risk individuals
- Yes, but it is not widely available
- No, it is not being utilized
- I'm not sure

4) What are the methods and imaging modalities available in primary care?
- CT scan
- Chest X-ray
- Pulmonary function test
- Sputum cytology
- Other (please specify)
- I'm not sure

5) Are there specific criteria or guidelines in use for imaging tests when there is suspicion of lung cancer?
- Yes, please describe (Kindly attach the guidelines or a direct link if available)
- No
- I'm not sure

B. **Diagnostic Phase in Specialized Healthcare**

6) How long does it typically take for a patient to receive a referral for a specialist consultation after their initial visit to a general practitioner?

- 1-2 weeks
- 2-4 weeks
- 4-6 weeks
- More than 6 weeks
- I'm not sure

7) How long does it typically take for a patient to receive a lung cancer diagnosis at the hospital after their visit to a specialized consultation?

- 1-2 weeks
- 2-4 weeks
- 4-6 weeks
- More than 6 weeks
- I'm not sure

8) For patients with non-small cell lung cancer (NSCLC), is extensive molecular genetic testing performed before treatment planning at your hospital?
- Yes, always
- Only for specific indications
- No, never
- I'm not sure

9) If yes, please specify what molecular genetic tests are performed for NSCLC patients:
   - EFGR
   - ALK
   - ROS1
   - PDL1
   - KRAS
   - BRAF
   - HER2
   - RET
   - MET
   - NTRK
   - Other:
   - I'm not sure

10) For patients with small cell lung cancer (SCLC), is extensive molecular genetic testing performed before treatment planning at your hospital?
   - Yes, always
   - Only for specific indications
   - No, never
   - I'm not sure

11) If yes, please specify what molecular genetic tests are performed for SCLC patients:
   - EFGR
   - ALK
   - ROS1
   - PDL1
   - KRAS
   - BRAF
   - HER2
   - RET
   - MET
   - NTRK
   - Other:
   - I'm not sure

12) What type of cases are discussed at MDT meetings?
   - All lung cancer cases
   - Only complex cases
   - Only cases with multiple treatment options
   - Only cases with local treatment options
   - Others (Please specify)
   - I'm not sure

13) What specialities are represented at MDT meetings ?
   - Oncology
   - Radiology
   - Pathology
   - Surgery
   - Pulmonology
   - Laboratory Geneticist
   - Nurse
   - Other
   - I'm not sure

14) During the diagnosis phase, is it standard practice to consult a surgeon?

- Yes, always
- In specific cases, please specify.
- No
- I'm not sure

15) a- What criteria are used to refer lung cancer patients for surgery? (Select one or multiple)
- Tumor stage and size
- Presence of metastasis
- Type of lung cancer
- Patient's overall health condition
- Patient's preferences and goals of care
- Other (Please specify)
- I'm not sure

16) Are there any local or national guidelines for the diagnostic phase of lung cancer care?
- Yes, and they are up-to-date
- Yes, but they are out-dated
- No

Kindly attach the guidelines or link if available

C. **Treatment Phase**

17) Does your hospital offer surgery for lung cancer patients?
- Yes
- No
- I'm not sure

18) If not, is there a difference in the referral practice for NSCLC and SCLC to a specialized center or hospital?
- Yes, please elaborate
- Depends on certain criteria, please elaborate
- No
- I'm not sure

19) What percentage of patients do not undergo surgery due to limited availability of surgical services?
- None
- Less than 10%
- 10-25%
- 25%-50%
- More than 50%
- I'm not sure

20) Who is eligible for surgery, and what factors determine the selection of patients for surgery?

- Eligibility determined by tumor stage
- Eligibility determined by patient health status
- Eligibility determined by the type of lung cancer
- Other factors (please specify)

21) What cancer treatments are currently available at your hospital for NSCLC and SCLC?

a- For NSCLC:
- Chemotherapy drugs
- Radiotherapy
- Targeted therapy drugs
- Immunotherapy drugs
- Others (please specify)

b- For SCLC:

- o Chemotherapy drugs
- o Radiotherapy
- o Targeted therapy drugs
- o Immunotherapy drugs
- o Others (please specify)

22) How many treatment lines are typically offered to lung cancer patients at your hospital?

a- For NSCLC:

- o A single line of therapy
- o Two sequential lines of therapy (eg. A combination of cancer treatments)
- o Three sequential lines of therapy
- o Four or more treatment lines (access to advanced therapy)

b- For SCLC:

- o A single line of therapy
- o Two sequential lines of therapy (eg. A combination of cancer treatments)
- o Three sequential lines of therapy
- o Four or more treatment lines (access to advanced therapy)

23) Is the neoadjuvant treatment guided by specific guidelines or individual medical decision-making?

- o Typically guided by specific guidelines.
- o Primarily based on individual medical decision-making.
- o Both play a role in determining neoadjuvant treatment.
- o It varies depending on the specific circumstances or healthcare provider.
- o I'm not sure.

24) What percentage of patients receive adjuvant treatment based on meeting the criteria for adjuvant therapy according to the postoperative stage?

- o Less than 25%
- o 25%-50%
- o 50%-75%
- o More than 75
- o Not sure

25) Are the reimbursement decisions for medications administered in the hospital determined locally or nationally?

- o Nationally
- o Locally (At hospital level)
- o Both, please specify
- o I'm not sure

26) For each medication type, specify where they are reimbursed from:

a- IV:

- o Hospital
- o National Institution
- o Insurance companies
- o Patient's private insurance
- o Other:
- o I'm not sure

b- Tablets:

- o Hospital
- o National Institution
- o Insurance companies
- o Patient's private insurance
- o Other:
- o I'm not sure

27) Do you believe that the reimbursement allows all patients at your hospital to have access to medically appropriate medicine? Please elaborate
   - Yes
   - No

D. Follow-up Phase

28) What methods are used in the follow-up of lung cancer patients?
   - Chest X-ray
   - CT imaging
   - PET imaging
   - Blood tests (e.g. tumor markers)
   - Physical examination
   - Other (please specify)
   - I'm not sure

29) Are there national recommendations for follow-up frequency for lung cancer patients?
   - Yes
   - No
   - I'm not sure

Attach guidelines

30) What is the duration of active follow-up in specialized care for lung cancer patients?
   - Less than one year
   - 1 year
   - 2 years
   - 3 years
   - 4 years
   - 5 years
   - More than 5 years
   - I'm not sure

31) What is the interval of the follow-up appointments during the designated period?
   - Every 3 months
   - Every 4 months
   - Every 6 months
   - Once annually
   - Other:
   - I'm not sure

32) Is follow-up continued in primary healthcare?

   - Yes, frequency ___ /years
   - No
   - I'm not sure

33) Is there a national quality register specifically for tracking lung cancer treatment outcomes:
   - Yes
   - No
   - I'm not sure

34) Do you follow the outcomes of the lung cancer patients (eg. mortality, survival rate) of your hospital:

   - Outcomes are discussed regularly and systematically in the hospital
   - Outcomes are measured, but the results are not regularly analyzed in the hospital
   - Outcomes are not systematically measured
   - I'm not sure
